# Molecular Characteristics of H6N6 Influenza Virus Isolated from Pigeons in Guangxi, Southern China

**DOI:** 10.1128/genomeA.01422-15

**Published:** 2015-12-03

**Authors:** Meng Li, Zhixun Xie, Zhiqin Xie, Sisi Luo, Liji Xie, Li Huang, Xianwen Deng, Jiaoling Huang, Yanfang Zhang, Tingting Zeng, Sheng Wang

**Affiliations:** Guangxi Key Laboratory of Animal Vaccines and Diagnosis, Guangxi Veterinary Research Institute, Nanning, China

## Abstract

Here, we report the complete genome sequence of an H6N6 avian influenza virus (AIV) isolated from a pigeon in Guangxi, southern China, in 2014. The eight RNA segment genes shared a high nucleotide identity (97 to 99%) with H6N6 subtypes of AIV isolated from ducks in the regions around Guangxi Province. The finding of this study will help us understand the ecology and molecular characteristics of H6 avian influenza virus in wild birds in southern China.

## GENOME ANNOUNCEMENT

Avian influenza A virus (AIV) is a single-strained negative-sense RNA virus, which can cause a variety of infections in avian and mammalian species. The H6 subtypes of influenza viruses are the most abundantly detected influenza virus subtype, and they have a broader host range than any other subtype (1). In the past decade, surveillance studies have revealed the existence of different subtypes of H6 viruses, which showed that most of the strains were isolated from ducks, chickens, and geese (2–6). Recent reports have detected the H7N9 viruses in healthy pigeons, and these viruses have once again come into the spotlight for their potential role as a bridge among species in the ecology of avian influenza (7, 8). However, the epidemiology and biological characterization of the H6 virus isolated from pigeons are still unknown in China.

We report here the complete genomic sequence of an H6N6 avian influenza virus strain, A/pigeon/Guangxi/161/2014(H6N6) (GX161), which was first isolated from pigeons in Guangxi Province, southern China, in 2014. All eight gene segments were sequenced by a DNA sequencing service company (TaKaRa). The sequences were assembled and manually edited to generate the final genome sequence, as in a previous study (9).

The full lengths of the polymerase basic 2 (PB2) and PB1, polymerase acidic protein (PA), hemagglutinin (HA), nucleoprotein (NP), neuraminidase (NA), matrix (M), and nonstructural (NS) genes were 2,341, 2,341, 2,233, 1,744, 1,565, 1,464, 1,027, and 890 nucleotides, respectively. Phylogenetic analyses showed that the HA and NA genes belonged to the same clade as H6N6 viruses currently circulating in China, such as A/duck/Guangxi/Gxd-7/2011 and A/duck/Fujian/7818/2007 (up to 97% nucleotide identity with the reference strains). The other six genes were found to be more similar to those of Chinese and Vietnamese H6N6 AIV strains (up to 99% nucleotide identity). Interestingly, all the reference strains were isolated from ducks in the different regions around Guangxi Province, and this result maybe also suggests that H6 subtype avian influenza viruses can transmit direct from ducks to pigeons.

The amino acid motif of the cleavage site between HA1 and HA2 was PQIETRG; this is a typical characteristic of the low-pathogenicity avian influenza virus (10). The pigeon GX161 strain has Q226 and G228 (according to H3 numbering) at the receptor-binding site in the HA protein, which was different with S228 detected in both swine and human H6 isolates; this result suggests that the strain has the ability to bind a sialic acid-2,3-NeuAc Gal linkage (11). The possession of 627Glu and 701Asp in the amino acid sequence of PB2 protein, which still has the characteristics of avian influenza viruses, so it was not be able to replicate in mammalian hosts (12). Positions 26, 27, 30, 31, and 34, which did not have an amino acid point mutation in the matrix 2 (M2) protein, showed that the GX161 strain is not amantadine resistant (13). This study will help to understand the molecular characteristics of H6 subtype avian influenza viruses in pigeons.

### Nucleotide sequence accession numbers.

The genome sequences of A/pigeon/Guangxi/161/2014(H6N6) have been deposited in GenBank under accession numbers KT267019 to KT267026.
